# Twenty-fourth Report of the McLean Asylum for the Insane

**Published:** 1842-04

**Authors:** 


ART. IV.—*Twenty-fourth Report of the McLean Asylum for the Insane.* By LUTHER V. BELL, M. D., Superintendent. Boston, 1842, p. 40,

We have read this document with great satisfaction. Like its predecessors, it is filled with important suggestions relative to the management of the insane. The statistical tables are not so copious as we would like to see—we believe them to be important elements for the formation of our opinions as to this or that place, or mode of treatment. Notwithstanding they are often abused, and made to convey more meaning than their elementary facts justify, yet they are the safest method by which we can approximate truth, in medicine and the other inexact sciences, as well as in the stricter sort; and we are very sure, that tables, founded upon the careful and conscientious observations of Dr. Bell, could not lead us astray.

The usual success of former years has followed the labors of Dr. Bell during the last year. In that period, he had 283 patients under his care: Of these, seventy-five recovered; twenty-nine were discharged stationary, twenty-four improved; two were unfit subjects, eleven died, and one hundred and forty-two remained in the asylum at the end of the year. Many of the improved were taken away by the impatience or economy of friends, and the stationary were discharged, for want of accommodations, to make room for the more hopeful. Even with these disadvantages of reckoning, we find sixty per cent. of all discharged were cured, which is a much larger success than the average of asylums has exhibited.

Dr. Bell, through his report, offers valuable considerations upon the treatment of insanity—upon the errors which common statistics unintentionally convey, for want of sufficient explanation of the minuter circumstances—and lastly, upon "the relative value of medical and moral means of treatment." Upon this last head, the views of this faithful and practised observer are peculiarly valuable.

"Each year that I have passed in this extensive field has served to diminish my confidence in an active medical treatment of almost every form of disease of the mind, and to increase my reliance upon moral means. No individual at the head of an insane institution would now think of combating any form of insanity, with the depletory and reducing means once regarded as indispensable. The practice of bleedings, violent purgations, emetics, vesicatories and derivatives, has passed away before the light of experience. A different and opposite mode of treatment by energetic sedatives, I am satisfied, is obnoxious to many objections, although far to be preferred to the first. The recoveries under their administration, occasionally most magical and most gratifying in appearance and for the present, as far as my observation and experience have extended, are neither so perfect nor so permanent as under a less decided course of measures. A *wise expectation* and a cautious use of medical agents to meet symptoms, comprise most of the aids that the pharmacopœia is capable of affording. But in relation to moral means, especially carried through as they can be only by the instrumentality of an appropriate institution, my annual experience has only exalted my confidence."

To carry out these views, it is necessary that the superintending physician should not only visit, but live in the asylum, live with and among his patients, watch every symptom and every movement, be ready for every emergency, and make his moral means as active as the varying course of his patient's diseases may require.

Under the head of moral means, Dr. B. includes, 1. Separation from friends and ordinary associations, and also the interdiction of visits, letters and the like. 2. Direction, including the application of such means as are adapted to the varying circumstances, developments, tastes and symptoms of the different patients. 3. Classification. 4. Occupation, with every variety of employment whether useful or amusing.

Dr. B. urges upon the friends of the lunatic, to become well acquainted with the nature and conditions of the insane asylum in which they wish to place their deranged patient; "to ascertain whether they can rely upon its faith, its skill, and its kindness;" to inform the lunatic clearly and distinctly of the object of his separation, of the character of the institution and of the nature of the confinement, and thus prepare themselves and him for a long separation, if the case should require it. Then, having made their selection of a hospital, in which they can trust their friend, and having determined to abide by the judgement of the officers as to his continuance, they should leave him, without distrust or intermeddling, in their hands. For this interference of impatient friends is, and has been, one of the greatest obstacles to the cure of the insane in private institutions, which have no power to retain their patients beyond the consent of friends abroad,

"We would again say to friends, do not bring your patient to us if you can do as well or better by him at home or elsewhere; do not bring him with the impression that you are to regulate his treatment, or that any peculiar knowledge you may have of his traits of character will enable you to decide, better than the institution, where he shall be placed in its classification, when or by whom he shall be seen, or when he is in a fit state to be removed. This asylum desires to receive no patients except on its own terms; it asks no patronage; it was established to confer not to receive benefits."

It would seem that the advantages of a residence in an asylum over one at home, for the insane, would need no further proof. But when we find that not an eighth of all the insane in the nation are sent to asylums, that new cases are perpetually occurring, and are suffered to become chronic, and therefore incurable, from neglect, we are convinced that the case needs further argument and illustration. We cannot urge this matter too earnestly home to every physician and citizen, that most cases of lunacy can be cured by early removal from home and friends, from habitual associations, to a proper asylum; and that on the contrary, most cases become hopelessly incurable, if not duly and seasonably attended to. The world, professional and non-professional, in its best condition, has not been made to feel the full force of this truth, and has, therefore, been negligent of this class of sufferers—No disease has been neglected like insanity. Derangement of the lungs, or of digestion, calls for and receives immediate and skilful attention; but derangement of the intellect or moral feeling, is strangely disregarded, or too often submitted to most quackish experiments. Hence we need to keep this subject continually before the people, until all are convinced of the necessity of early attention to the insane. Although so much has been written and published upon this in Massachusetts, still Dr. Bell finds it necessary to urge it again; and if it be needed there, where the matter has been so long discussed, it will not be out of place here, in this valley, where we have nearly four thousand lunatics and idiots abroad, and less than five hundred in our asylums.

The experience of all ages and all countries coincides in its importance, so that the general statement is fully warranted that patients seized with insanity and removed to an institution usually recover; while those treated at home generally do not. Not unfrequently removal alone has appeared adequate to effect an entire change of the diseased mental action.

If a patient be irritable, excited and violent, experience shows that he will not submit to friends as readily as to strangers. If feelings of hatred and vindictiveness exist, they will be probably directed towards those in whom he ought most to confide. If a master of a family and accustomed to direct, he will not readily and without reason as it appears to him abdicate his authority. If a child in his father's house, his insane sagacity will at once show him that he need not yield peaceably the government of himself, where the tender feelings of an afflicted family will deny the firmness to insist. If restraint becomes indispensable, a wide difference will be found in its application as administered by new hands, and by those whose experience has taught them that mildness and decision are not incompatible, and that strength and violence may distress and aggravate, but do not often calm the maniac. There should be, in fact, as mnch difference in a well regulated institution in the character and extent of physical force necessary to control a dangerous patient, as applied by experienced and well disciplined attendants, and the ordinary aids called to effect the same objects in families, as there is between the leathern mittens and secure rooms of an asylum, and the chains, strait waistcoats and cages, which are deemed indispensable in furious cases without the walls of a hospital.

If hallucinations and delusions constitute the leading features of a case it is almost impossible for a patient to be removed from conversations and associations continually renewing their strength in the mind, while at home or under the guidance of those unskilled. Friends and acquaintances can hardly avoid attempting the effect of argument, remonstrances, appeals to the feelings, or even threats. It is natural that those whose experience is limited to a single case, should do so; while they who have been taught by experience the futility of such attempts, will not aggravate and excite a diseased point with continual meddling."

No part of an asylum is without its influence over the insane. It is not a matter of indifference how it is organized—nor who are employed to superintend, direct, or assist, in its administration. The superintending officers should have the highest intellectual and moral cultivation. Of their duties and responsibilities Dr. Bell says, "it can hardly be expected of the writer to touch." To those who knew him it was not necessary; for his life and practice were the argument and the explanation of these matters. But of the subordinate aids, he is more free to speak. "My present design is to urge the indispensable value of an elevated, intelligent, and conscientious corps of assistants, limited in number only by the extent to which they can be profitably and usefully employed. Had an institution a Pinel or an Esquirol for its head, with as perfect a suite of architectural arrangements as the ingenuity of man has devised, and were obliged to depend upon a few ordinary *keepers*, its character and its results must inevitably fall far below the practicable maximum."

"In cases of considerable duration, the prospect of benefit will depend upon the amount of persevering, well-directed labor which is bestowed. To secure such labor, an honorable appreciation and an adequate remuneration are requisite. In this country, such services as those of the best attendance upon the insane, can only be had by a liberal expenditure of money. As so much aid is required in the full employment of moral means, it is manifest that a proper keeping and care of the insane, must always, as compared with the support of those of sound mind, be expensive."

"The assistants in the care of the insane must not only be well selected and well compensated, but well instructed. There are natural gifts of person, address and temper, which are not to be disparaged, but beyond these there is a tact, an aptitude, a discrimination in managing the insane, which can only be acquired by observation and practice, after being put upon the right track. Some persons with apparently adequate gifts of nature, never are able to acquire this art."

"The systemwhich has always been pursued here, is that of elevating the office of assistant. Neither the term *keeper* or *servant*, has ever been known in the intercourse of attendant and patient. The latter has always been led to look upon the former as a friend and equal." . . . . "The test question by which the character and fitness of every male and female caretaker of the insane should alone be tried is, whether under like circumstances, the director would desire his wife, children or parents, to be submitted to the same hands."

Classification is of great importance in the McLean Asylum, and the numerous buildings, and diversified arrangement of them, enable it to carry this to great extent. Thus not only injury is avoided, but great curative advantages are obtained.

"It will readily be conceived that to place a wild and frantic maniac in an apartment with a refined and timid individual, whose sole deviation from soundness is one false or delusive idea, or to permit a demented epileptic lost to all sense of decency and propriety, to sit at table with one whose intellect perhaps is unchanged, but whose affective sentiments alone are diseased, would be alike inconsistent with the happiness or recovery of the party capable of suffering." . . . . . "There is a nice adaptation of intellects, of social position, of cultivation of mind and manners to be arranged in consociation."

"Experience shows that the very highest curative advantages are effected by the mutual action and attrition of diseased minds upon each other. Emulation, forbearance, self-respect, benevolence, diversion of diseased ideas or sentiments into new and natural channels, are all capable of being secured by a proper association of different individuals."

Occupation, varied and well adapted, is still found beneficial in this institution. Dr. B. adds to his former testimony the confirmation of another year's experience of its good effects on the insane.

"The general law, both as regards the curative treatment or custodial comfort of the patient, is constant but varied occupation of body and mind. To ensure this, requires every opportunity and aid of labor and amusement. The more perfect the system of an institution, the more ample will be the provision to secure these ends. The different education, tastes and pursuits of individuals, will render it impossible to subject all to the same course in this respect. To one, agricultural or horticultural avocations; to another, mechanical employments; to a third, the fine arts or some form of busy idleness must be adapted."

Nor are these all the moral means of treating insanity.

"There are still other adjuvants, such as judicious counsel, explanations, and at a proper time even reasonings, a proper participation in the services of religion, and a regulated intercourse with the sane, all of which are of the highest utility."

We have been thus free in our extracts from this report, because this asylum is one of the oldest and best in the country, and has taken the lead in most of the improvements of the present age in the management of the insane; moreover, the full and elaborate reports of its able superintendent have described the principles and details of practice there, and we are glad to give his views a wider diffusion in the west, than they could have through his report alone.

The following extracts from "the directions for admission" are as applicable to the patients, and as useful to the asylums in the west, as they are to the McLean, from which they emanate.

"It is desired that no case (unless certainly of the most recent duration,) should be sent to the institution, unless it is determined and arrangements made, to afford a fair trial. No time short of six months can justly be considered a fair trial, and to place a patient afflicted for many months here with the determination to remove him at the expiration of a single quarter, is an injustice to the Asylum, and to the general cause of the insane.

It is presumed that the friends and guardians of every patient placed here, are satisfied that they can rely upon the skill, kindness and integrity with which he will be treated, and that they can do no better with their charge. Having arrived at this conclusion, as they should do, before committing their friend to its cares, it will be recollected as a part of the covenant, that the discretion of the Superintendent is to decide in every case upon the patient being seen by friends, or acquaintances or strangers, or visits being absolutely interdicted."

This Asylum is under the care of a Board of Trustees, who, by their sub-committees, visit the institution weekly. This duty of visitation has not been omitted for twenty-four years, with the exception of a single week. This is a guaranty for the faithfulness of these officers to their charge, and that no neglect or abuse, in those who have immediate care of the patients, can escape notice. E. J.

